# An Unusual Presentation of Spontaneous Rupture of Dermoid Cyst

**DOI:** 10.7759/cureus.21976

**Published:** 2022-02-07

**Authors:** Isaac Alsallamin, Ryan Choudhury, Francisco J. Somoza-cano, Austin Makadia, Mythri Mudrieddy, Anastasiia Weiland, Ameed Bawwab, Afnan Alsallamin, Faris Hammad, Kanchi Patell, Abdul Rahman Al Armashi

**Affiliations:** 1 Internal Medicine, St. Vincent Charity Medical Center, Cleveland, USA; 2 Internal Medicine, St. Vincent Charity Medical Center, cleveland, USA; 3 Internal Medicine, Northeast Ohio Medical University, Cleveland, USA

**Keywords:** general neurosurgery, neurosurgery, intracranial tumours, primary headache disorder, intracranial dermoid cyst, lumbar disc herniation surgery, traumatic csf leak, meningitis pain, dermoid cysts, headache disorders

## Abstract

Intracranial dermoid cysts are exceptionally rare tumors. Interestingly, this condition has a low mortality rate but a high morbidity rate due to its numerous complications. We report a case of a 62-year-old man who presented with a headache and was found to have a ruptured dermoid cyst, complicated with the dissemination of lipid droplets within the subarachnoid space.

## Introduction

Dermoid cysts comprise a large category of site-specific cysts. They are embryonic in origin and can be located in various organ systems, including skin, reproductive or nervous systems. These cysts can contain different structures like hair, fat, nails, cartilage, and bone. Dermoid cysts represent 0.04%-0.6% of all intracranial tumors [1].

A rupture of a dermoid cyst within the central nervous system is an uncommon complication which may present with headache, seizures, or a mass effect. This condition has low mortality but significant morbidity, giving its various manifestations [2-5]. 

Diagnosis is challenging and MRI is the best imaging modality that demonstrates high intensity on T2-weighted imaging due to high fat content [3-5]. Treatment is usually conservative, but surgical intervention of severe complications or refractory symptoms is an alternative approach with a very high success rate and low recurrence [1,2,6,7,8].

## Case presentation

**Figure 1:**
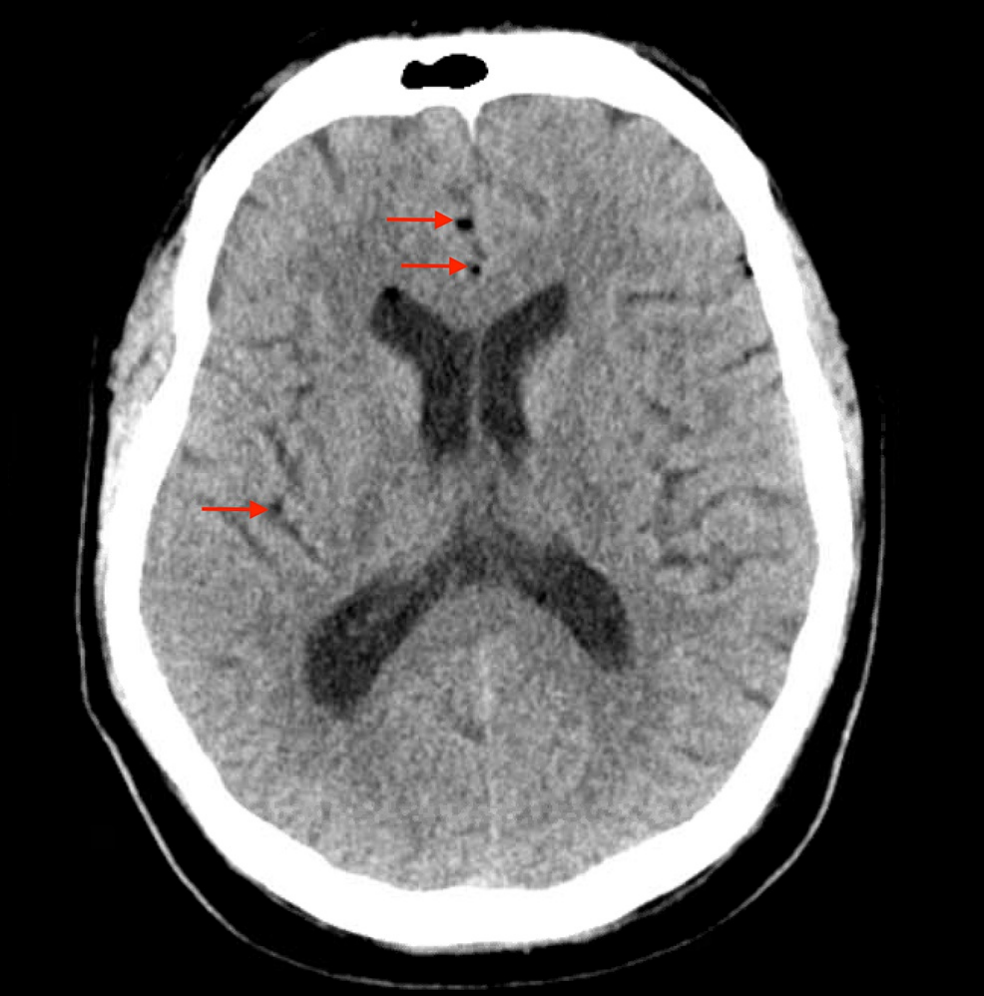
Brain CT scan without contrast, pneumocephalus Red arrows show gas-like foci, pneumocephalus.

A 62-year-old right-handed Caucasian man presented to the hospital with a headache and back pain complaint after a recent L5-S1 redo discectomy with fusion secondary to lumbar stenosis. His past medical history included hypertension, type 2 diabetes mellitus, recurrent major depressive disorder, hypothyroidism, gastroesophageal reflux, anxiety, alcohol use, lumbosacral herniated nucleolus pulposus, and previous L5-S1 discectomy three years prior to the date of presentation. In addition to the back pain, the patient also reported that when riding home in the car on the day of discharge, he developed a sudden intense circumferential pressure like headache that was 8 out of 10 in severity. He endorsed phonophobia, but no photophobia, not radiated, no nausea, or vomiting. There were no alleviating or aggravating factors, and the patient tried taking Tylenol for the headache with no relief. He mainly laid in his bed after the initial surgery until he presented to the hospital with worsening back pain. On the patient's return to the hospital, he underwent a CT of the lumbosacral spine, demonstrating a superficial fluid collection that likely represented a seroma versus a hematoma. The following day, he underwent incision and drainage of the fluid in the operating room under general anesthesia without any complications. Fluid was blood-tinged and sent for culture analysis. At this time, the patient reported a headache as his primary symptom, and the neurology team was requested to assess the patient. A CT of the brain without contrast was performed and demonstrated multiple small foci of a low-intensity signal which were thought to be pneumocephalus, given the recent history of spinal surgery (Figure 1).

**Figure 2:**
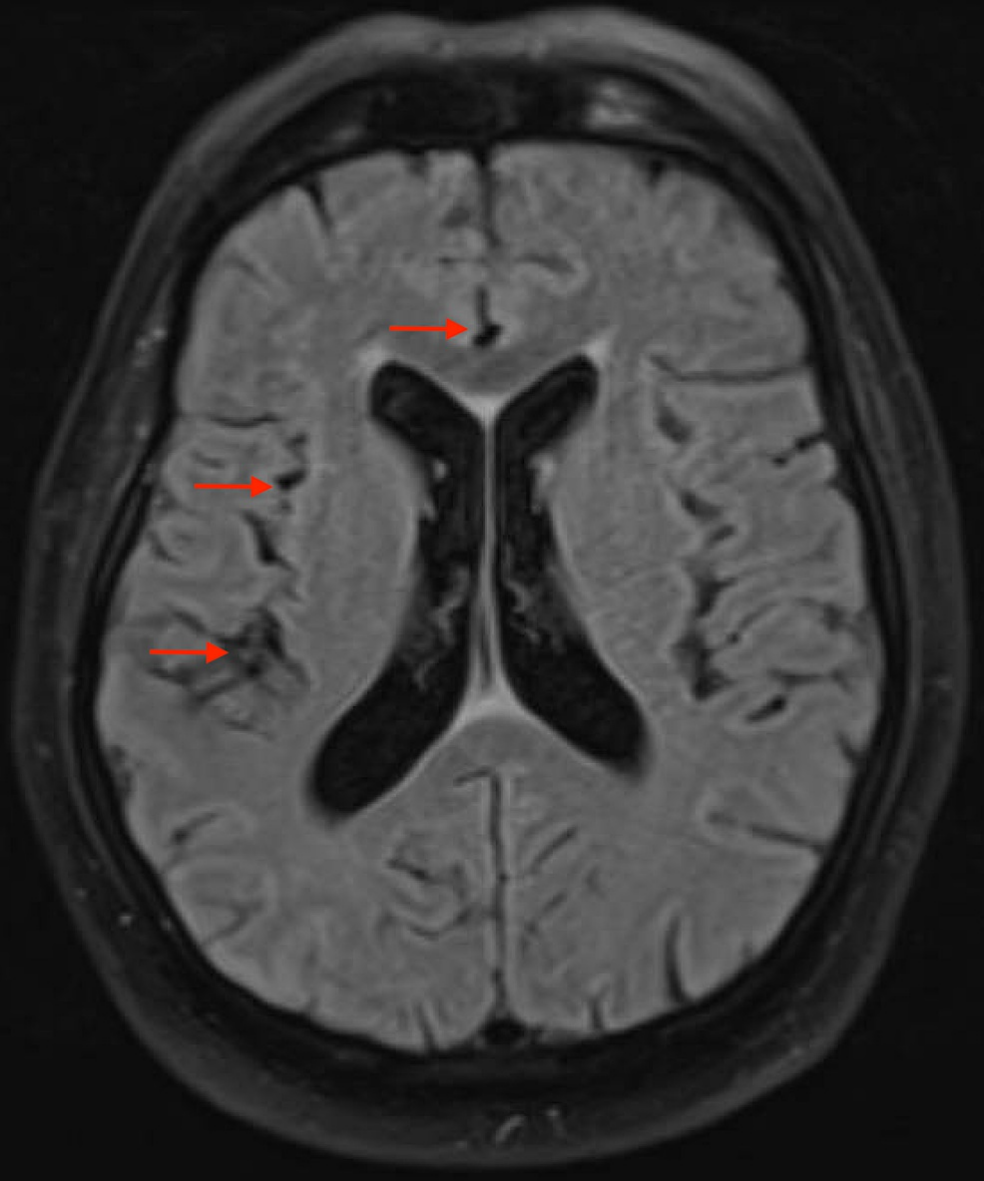
Brain MRI FLAIR axial view Brain MRI fluid attenuation inversion recovery (FLAIR): hypodense area; Red arrows: fat drops; Bright white area: ventricular ependymal enhancement and hyperintense areas.

**Figure 3:**
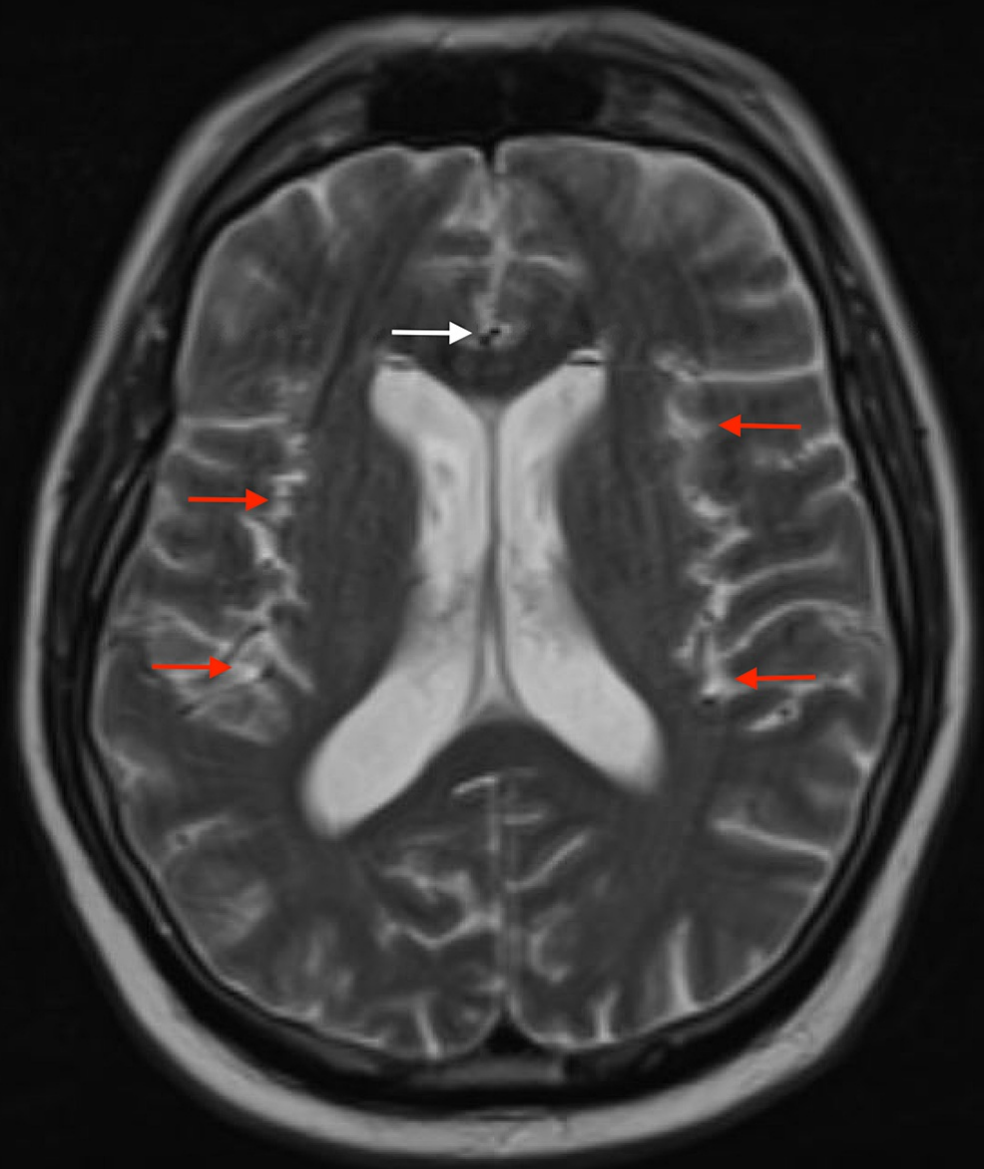
Brain MRI T2 axial view Brain MRI axial T2: hyperintensity signals with heterogeneous appearance; Red arrows show hyperintensity signals: fat drops; White arrow shows hypodense signals at frontal area and represent fluid signal.

**Figure 4:**
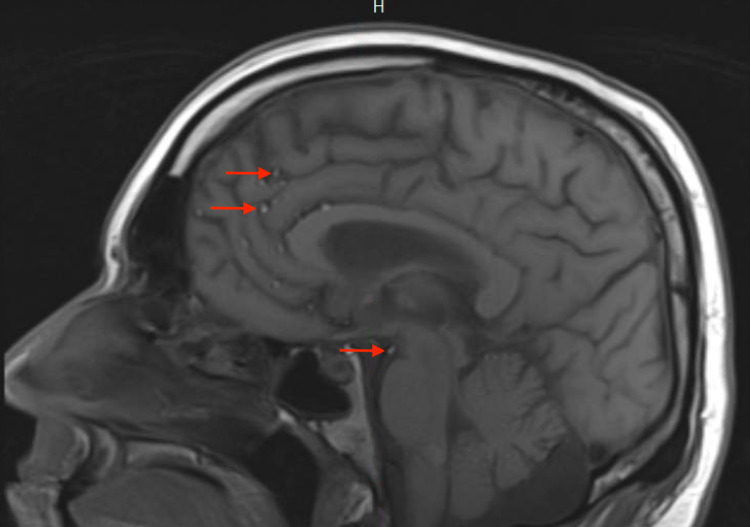
Brain MRI, sagittal T1 Brain MRI T1 shows hyperintensity signal; Red arrows: fat drops in subarachnoid space.

Anesthesia and operative reports were reviewed from the redo discectomy and indicated that the patient received general anesthesia but never received any spinal anesthesia in the preceding period. The dura was not entered during the surgical procedure, and there was no leak of cerebrospinal fluid with Valsalva maneuvers in the operating room prior to closure. Given these reports, the CT head was reviewed with the radiologist again as no source for pneumocephalus was identified. Using the lung window view on the CT head, the foci suspicious for pneumocephalus did not resemble free air and appeared more similar to the enhancement seen with lipid accumulation. An MRI of the brain was then recommended to assess the etiology of these foci in the cerebral spinal fluid. The MRI demonstrated the same multiple foci within the cerebrospinal fluid, and their enhancement on diffusion-weighted imaging and T2 were consistent with lipid accumulation without any identifiable cystic lesions (Figures 2-4).

At this point, the patient was diagnosed with a likely rupture of an intracranial versus intraspinal dermoid cyst. Conservative management with analgesics opioids was given in the postoperative setting, and non-steroidal anti-inflammatory drugs resulted in headache improvement. After a couple of days, the patient was discharged home after improving his condition with home therapy and follow-up. He was instructed to immediately go to the emergency room for evaluation for any new neurologic symptoms such as seizure, numbness, weakness, or uncontrollable headache. He made a full recovery thereafter and he was scheduled for serial follow-up imaging studies.

## Discussion

Dermoid cysts are benign tumors that can present in several distinct anatomical locations. Intracranial dermoid cyst symptoms are specifically related to mass effect, headache, and very rarely, as spontaneous rupture [1,2]. Dermoid cysts represent 0.04%-0.6% of all intracranial tumors [1]. Ruptured intracranial dermoid cyst is a rare complication, as the dermoid cyst has thick capsules. Most cases are spontaneous rupture, as traumatic ruptures are exceedingly rare [3-5]. Ruptured intracranial dermoid cyst, present with headache, seizures, and mass effect. Symptomatology depends on the cyst’s location; it may rupture into the ventricular, subarachnoid or intracerebral space [3,4]. CT scans may show multi-hypodense foci or gas-filled structures or foci.

Brain MRI is recommended to diagnose and prompt early recognition of a ruptured dermoid cyst as MRI will give better details about the content of these cysts. Advanced MRI imaging typically recognizes ruptured dermoid cyst as the following: scattered hyperintense floating fat drops will be seen on the non-dependent ventricles and/or subarachnoid space; these lesions look hyperintense on MRI T1-weighted images. Hyperintense means bright white; hyperintensity depends on the fat content, the more fat the more hyperintense. On T2-weighted images, lesions will be hyperintense or heterogeneous depending on the fat content. On fluid attenuation inversion recovery (FLAIR) imaging, lesions look bright white with fat content on hypodense attenuation; CSF-like fluid depends on the fluid and fat content of the lesions. Pial and ventricular ependymal enhancement may be seen after gadolinium contrast. MRI early diagnosis and management improve the chance for better outcomes and timely surgical intervention if needed [3,6,9]. 

Treatment is usually conservative management. Occasionally, dermoid cysts require surgical intervention, especially when large or complicated. Partial or complete surgical resection of complicated hydrocephalus cases required shunt surgery. Conservative management is not limited to pain control, anti-nausea and anti-vomiting medications [1,10,11]. A trial of steroids to avoid and treat chemical-aseptic meningitis is a transient complication most of the time [1,2,5,9,10]. 

Our case presented as a spontaneous rupture of an intracranial dermoid cyst, diagnosed with MRI, after eliminating other causes since our patient recently had lumbar spine surgery; investigations ruled out dural injury, CSF leak, or infection. Our patient responded appropriately to conservative management. Severe complications may require surgical intervention. Hydrocephalus, recurrence, or mass effect require immediate evaluation by neurosurgery. Seizure is another serious complication that mandates regular follow-up. 

A close monitoring for new emerging symptoms and signs like headache, neck stiffness, photophobia, etc. that mimics meningitis due to meningeal irritation, is highly suspicious for ruptured intracranial dermoid cyst, or its complications; this mandates a full sequence brain MRI which is the golden modality for diagnosis [5,10,11].

We describe the case of a patient who developed a headache during the post-operative period of lumbar surgery. The timing of his symptoms and the relevant clinical imaging indicate that he likely developed a spontaneous rupture of a dermoid cyst somewhere along his intracranial or intraspinal tract. He was managed with conservative therapy and scheduled for follow-up with neurology after surgery. Three weeks after discharge, the patient continued to report low-level headaches occurring daily with an intensity of 2 out of 10. The pain improved with acetaminophen, and the patient followed up with his doctors in the post-operative period. Our patient never presented on follow-up at the neurology clinic, as his headache improved significantly, and he never repeated brain MRI on his own accord.

## Conclusions

Our case highlights a spontaneous intracranial cyst rupture, a rare complication of dermoid cysts. Early detection and treatment can prevent serious complications such as obstructive hydrocephalus. It also illustrates that close monitoring for new symptoms is vital, to prevent life-threatening complications and referral for s surgical interventions if required. 
